# Pre‐ and post‐operative voice therapy (PaPOV): Development of an intervention for patients with benign vocal fold lesions

**DOI:** 10.1111/1460-6984.12771

**Published:** 2022-09-01

**Authors:** Anna White, Paul Carding, Vicky Booth, Pip Logan

**Affiliations:** ^1^ Centre for Rehabilitation and Ageing Research, Academic Unit of Injury, Inflammation and Recovery Sciences, School of Medicine University of Nottingham Nottingham UK; ^2^ Faculty of Health and Life Sciences Oxford Institute of Nursing, Midwifery and Allied Health Research Oxford UK

**Keywords:** benign vocal fold lesions, intervention development, pre‐ and post‐operative voice therapy, triangulation, voice disorder

## Abstract

**Background:**

Pre‐ and post‐operative voice therapy may improve voice and quality‐of‐life outcomes for patients undergoing phonosurgery to remove benign vocal fold lesions (BVFLs). However, what constitutes voice therapy in this population is poorly described, resulting in a poor evidence base, lack of clinical guidelines and unwarranted variation in management. In order to develop the evidence base, a robust, iterative process of intervention development work should precede feasibility testing and effectiveness studies.

**Methods & Procedures:**

Guidance for developing complex interventions, drawing on evidence, theory and modelling, was used to inform the development of a pre‐ and post‐operative voice therapy intervention entitled ‘PaPOV’. Data from four sources of evidence were synthesized using a published triangulation protocol. Data from a systematic review, national survey of current practice, expert interview study, and patient and public involvement conversations were used to populate a triangulation matrix, outlining components of a PaPOV. Data were coded to reflect areas of agreement, dissonance and silence with each component of the intervention. Based on this evidence, an assessment of convergence for each intervention component could be made.

**Outcomes & Results:**

In total, 61 components of the PaPOV intervention were explored. Of these, 27 were categorized as having stability of consensus according to a priori criteria. A total of 34 failed to meet the criteria. This was more frequently due to silence (27) rather than dissonance (seven) in the data. By evidencing areas of agreement and stability of consensus across data sources, the validity of individual findings has been enhanced. Furthermore, the study has exposed specific areas of the intervention that lack consensus and require exploration through further intervention development studies.

**Conclusions & Implications:**

This systematic triangulation process has contributed to the development of a PaPOV intervention for patients with BVFLs. Exploration of specific components relating to the intervention will allow outstanding questions to be answered in preparation for feasibility testing.

**WHAT THIS PAPER ADDS:**

**What is already known on the subject**

- BVFLs cause dysphonia by preventing vocal fold closure, impacting on vibratory characteristics and increasing compensatory muscle tension. Management for these patients is variable with them being offered phonosurgery, voice therapy, pharmacological management or a combined approach. Pre‐ and post‐operative voice therapy may improve both voice and quality‐of‐life outcomes. This patient group has unique complexities when considering voice therapy, including surgical preparation, wound healing and epithelial mobilization.

**What this paper adds to existing knowledge**

- This study uses a robust triangulation process to synthesize current evidence and patient experiences in order to inform the development of a PaPOV. It outlines some of the key components and considerations when delivering pre‐ and post‐operative voice therapy to adults with BVFLs. Furthermore, it serves as a methodological example for intervention development in complex interventions, highlighting key guidance and recommended processes for developing and evaluating complex interventions.

**What are the potential or actual clinical implications of this work?**

- The 61 components discussed as potential ‘ingredients’ for a PaPOV enable clinicians to reflect on key considerations when planning and delivering voice therapy to adults with BVFLs. This study highlights the pitfalls both clinically and in research of failing to describe interventions adequately and the benefits of using accurate, specific and agreed terminology in clinical practice, such as that outlined in the Rehabilitation Treatment Specification System (TRSS).

## INTRODUCTION AND BACKGROUND

Benign vocal fold lesions (BVFLs) cause dysphonia by preventing vocal fold closure, impacting on vibratory characteristics and increasing the likelihood of compensatory muscle tension. Patients with BVFLs may be offered surgery, voice therapy or pharmacological management. There is some evidence that voice therapy delivered in addition to phonosurgery may improve both voice and quality‐of‐life outcomes (Ju et al., 2013; Tang & Thibeault, 2017). However, there is no clear understanding of what constitutes voice therapy for patients with BVFLs who are undergoing surgery, and in practice this has led to unwarranted variation in management and resource allocation.

Voice therapy is a highly complex intervention involving multiple interacting and overlapping elements. Voice therapy interventions typically comprise a combination of indirect (information, education and advice) and direct (exercises) therapy (Gartner‐Schmidt et al., 2013) with individual tailoring according to predisposing, precipitating and perpetuating factors. Clinical sensitivity and judgement based on an ongoing assessment of the patients’ presentation and joint patient clinician discussions leads to individualization of voice therapy interventions. However, amongst the complexity and tailoring of these interventions, there are also components and considerations that are common to all rehabilitation interventions (Hart et al., 2019). Descriptions of rehabilitation interventions have been likened to a ‘black‐box’ phenomenon (Whyte & Hart, 2003) where treatments within it are poorly defined with respect to their characteristics and active ingredients (Desjardins et al., 2017; Hart et al., 2019; Ruotsalainen et al., 2007; Speyer, 2008). Voice therapy interventions have been recognized as failing to use specific, unique terminology resulting in treatments which may offer the same or similar intervention, framed in different terminology (Van Stan et al., 2021). There are repeated calls to describe the active ingredients and mechanisms of change in voice disorders research (Bos‐Clark & Carding, 2011; Patel et al., 2011; Pedersen & McGlashan, 2012; Schindler et al., 2012). Without this, the establishment, dissemination and synthesis of evidence based practice cannot occur (Van Stan et al., 2021).

Developing and evaluating complex interventions involves multiple phases, each impacting, informing and directing the next (Craig et al., 2008). Guidance from the Medical Research Council (MRC) emphasizes the importance of first examining the best available evidence and undertaking appropriate theory and modelling before pilot and feasibility testing are planned (Craig & Petticrew, 2012; Skivington et al., 2021). Preliminary evidence has highlighted that numerous factors influence pre‐ and post‐operative voice therapy (White & Carding, 2020) with high levels of variability and tailoring (Hart et al., 2019; White, 2020; Whyte & Hart, 2003). However, it has also exposed common considerations and components with this population (White & Carding, 2020). There is a substantial theoretical basis for a pre‐ and post‐operative voice therapy intervention (PaPOV) based on models of wound healing (Branski et al., 2005, 2006; Kaneko et al., 2017; Keylock et al., 2008), pre‐habilitation (Cantu & Steffe, 2013), motor learning (Bergan, 2010; Wenke et al., 2021, 2014) and behaviour change (Govender et al., 2017; Michie, 2014; Michie et al., 2013), but their application to this population has not been fully explored. In this context, the MRC guidelines emphasize the need to document intervention development work to avoid research waste, encourage a robust methodological approach, and improve understanding of the practical applications. Triangulation methodology is applied here to synthesize data from four sources of intervention development work which was undertaken to explore the components of a PaPOV for adults with BVFLs.

Triangulation as a research methodology explores convergence, complementarity and dissonance in data (Erzerberger & Prein, 1997). This in turn enhances the validity of the research by increasing the likelihood that research findings and interpretations will be credible and dependable (Lincoln & Guba, 1985). Triangulation of different data sources allows an issue of interest to be examined in a multidimensional manner (Farmer et al., 2006), thereby promoting a more comprehensive understanding of a phenomenon (Heale & Forbes, 2013). *Convergence* (or agreement) of findings leads to a definitive set of conclusions. *Complementary* findings add depth and increase the validity of findings through the process of verification (Heale & Forbes, 2013). Contrastingly, triangulation may identify *dissonance*, which highlights contradictory findings. Dissonance can be beneficial if it leads to further exploration of a phenomenon and a more nuanced understanding (Miles, 1994). Triangulation can be undertaken between research methods (e.g., interviews, focus groups and document analysis), data sources (e.g., interviews with professional versus patients), theoretical concepts and/or between investigators/researchers. Farmer et al. (2006) propose a detailed triangulation protocol to document each step and ensure transparency and replicability of the methodological process.

### Study aims

This study aimed to identify areas where agreement exists relating to a best‐practice PaPOV for adults with BVFLs. Triangulation was performed in order to:

- Describe a PaPOV intervention according to the TIDieR framework (Hoffmann et al., 2014) and the Rehabilitation Treatment Specification System's (RTSS) classification for voice therapy ingredients (Van Stan et al., 2019).
- Populate a triangulation matrix by identifying instances of agreement and dissonant findings in sources of evidence.
- Highlight areas of continued silence within the data sources.
- Identify where further interrogation, is required, as a result of dissonance or silence regarding a component of the intervention.

## METHODS

### Identification of data sources

Four sources of evidence were included in the triangulation process:

- A systematic review of pre‐ and post‐operative voice therapy for BVFLs, including 35 studies (White et al., 2021).
- A qualitative interview study with expert clinicians (*n* = 10) (White & Carding, 2020).
- A national survey of current practice (*n* = 69) (White, 2020).
- Patient and public involvement (PPI) from *n* = 5 patients with lived experience (face to face, virtual and email correspondence).

All sources of evidence contributed to an overarching research question: What are the key components of a best practice PaPOV for adults with BVFLs? However, each data set had differing purposes, individual research questions and limitations on the extent to which the overarching question was answered. For example, interview data specifically explored factors influencing the intervention, the systematic review data focused on components of the intervention, and survey data included content on some but not all components of the intervention. There was a mixture of qualitative and quantitative data within the included evidence and multiple methodologies. Therefore, according to Denzin's (2009) classification of triangulation methods, both methodological and data source triangulation techniques were employed.

### Triangulation protocol

Triangulation of data sets was undertaken following a protocol described by Farmer et al. (2006), which includes a six‐step process: (1) sorting, (2) convergence coding, (3) convergence assessment, (4) completeness assessment, (5) researcher comparison and (6) feedback. Minor modifications were made with the omission of step 5 as appropriate to the scope of the study (Table 1).

**TABLE 1:** Triangulation protocol steps taken

**Step**	**Activity**
1. Sorting	Sort findings from each data source onto segments that address the research question (items from the TIDieR framework are broken down into components of an intervention)
2. Convergence coding	Review all raw data, themes and findings from included data sets to identify where:
Agreement	there is agreement between a data set and proposed intervention component
Silence	the data set does not offer insights into the intervention component
Dissonance	there is disagreement within a data set or between a data set and the intervention component
3. Convergence assessment	Review all compared segments to provide a global assessment of the level of convergence. Document where further interrogation of a component is required
4. Completeness assessment	Compare the nature and scope of the topic for each data set to enhance the completeness of the united set of findings
5. Feedback	Feedback triangulated results to research team and patient and public involvement (PPI) members for review and clarification

*Source*: Based on Farmer et al. (2006).

#### Step 1: Sorting (development of the triangulation matrix)

**FIGURE 1:**
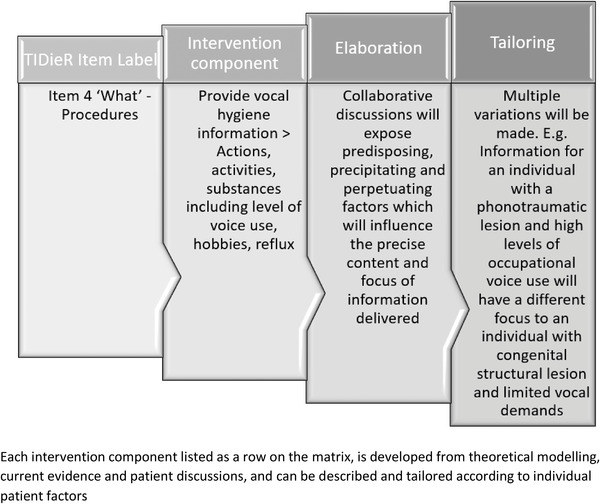
Context and tailoring associated with each intervention component

Sorting of the data sets involved the development of a triangulation matrix (see Appendix A in the additional supporting information). In accordance with intervention development guidance (Eldridge et al., 2016b; Skivington et al., 2021), the components of PaPOV were described according to the TIDieR framework (Hoffmann et al., 2014), with nine headings: (1) brief name, (2) why (rationale, theory and goal), (3) what (materials), (4) what (procedures), (5) who provided, (6) how, (7) where, (8) when and how much and (9) tailoring (see Appendix B in the additional supporting information). These headings were subdivided into suggested components based on the existing literature on wound healing, exercise physiology, management of muscle tension dysphonia, PPI views, and discussions with the study team with additional expertise sought from surgical colleagues. Procedures (item 4) were described according to voice therapy ingredients standardized in the RTSS (Van Stan et al., 2021). This framework proposes that any rehabilitation treatment can be characterized and classified according to its targets, ingredients and mechanisms of action (Hart et al., 2019) and has been specifically considered in relation to voice therapy, allowing unique targets and ingredients to be standardized (Van Stan et al., 2019). All ‘ingredients’ or components of the intervention were listed in rows on a triangulation matrix. Figure 1 shows an example of an ingredient label with further explanation and elaboration to illustrate the considerations and complexities behind each brief label provided. Data quotes were also captured to allow examples of convergence or dissonance to be illustrated clearly. The final matrix was reviewed by an active PPI group with lived experience of voice disorders to ensure applicability and acceptability to the patient group in question.

#### Step 2: Convergence coding

Convergence coding involved reviewing the raw data from all sources to identify instances where a data set gave insight related to any component of the intervention. Convergence coding was undertaken by the lead author and reviewed by two further members of the study team. Discussion between team members was used to resolve instances of ambiguity or uncertainty. Specific examples that supported or explained a particular component were documented. A convergence coding scheme (Table 1) was then applied, first to determine *agreement* between the four data sets on the essence, meaning and prominence of each of the components of the intervention, then to identify instances of *dissonance* and *silence*. Agreement was coded where there was evidence in a data set to support the inclusion of a listed component in the intervention. Dissonance was coded where evidence in a data set had conflicting meaning or recommendations relating to the inclusion of a component of the intervention. Finally, silence was used to highlight where a data set had no significant contribution regarding the specified intervention component. All four data sets were reviewed against each component of the intervention and coded on the triangulation matrix. Examples from the raw data were used to illustrate and justify decisions.

#### Step 3: Convergence assessment

The completed triangulation matrix was reviewed to assess the level of convergence present in the data sets analysed. This took into consideration the meaning and prominence of examples that supported inclusion of an intervention component. Decisions on the stability of the consensus across the four data sets determined whether a component was accepted into the intervention or if further exploration and interrogation was required (Table 2). If all four data sets were coded with agreement on a component of the intervention, this constituted full convergence and consensus for inclusion of the component in the PaPOV intervention. Dissonance in one or more data set pointed to the need for further exploration. Where silence existed in two or more data sets, consensus stability was not achieved, and further exploration was warranted.

**TABLE 2:** Convergence assessment: decisions on consensus stability

**Convergence assessment finding**	**Stability of consensus achieved**
All data sets coded with ‘agreement’	✓
Agreement in two‐thirds published data sets with no dissonance	✓
Dissonance across any data set	×
Silence in two or more data sets	×

#### Step 4: Completeness assessment

Completeness assessment involved comparing the four sets of results to highlight the similarities and unique contributions that each data set brought to the research question. The aim of this step was to ensure that there was completeness in perspective and in the ways in which the intervention components were represented by examples from the data sets (Farmer et al., 2006).

#### Step 5: Feedback

Throughout the process of triangulation, decisions were shared and discussed with three members of the research team. This was important to ensure reflexivity on decisions made, to allow comments, feedback and discussion around the process. PPI discussions were used to inform the development of the matrix and findings of the analysis were shared members of the PPI group, who confirmed their agreement with findings and with proposed next steps in the intervention development.

## RESULTS

### Sources of evidence

The nature of the four primary sources of evidence were as follows:

- A systematic review of PaPOV for BVFLs (White et al., 2021). The aim of this systematic review, comprising 35 studies, was to consider the content, timing and intensity of the PaPOV delivered to participants who were undergoing phonosurgery for BVFLs where the primary aim of the study was to evaluate voice and/or voice‐related quality‐of‐life outcomes following phonosurgery. This systematic review identified information pertaining to the content of voice therapy (indirect and direct), the timing (pre‐operative, immediate post‐operative care and longer post‐operative rehabilitation), intensity and dosage of exercises (including home exercise practice), and length and frequency of voice therapy sessions.
- A UK Survey of Current Practice of pre‐ and post‐operative voice therapy (White, 2022). Data from 69 respondents with a range of experience were included. Questions related to the broad content of the intervention and to the timing, but lacked depth of discussion on these elements. The survey used a combination of multiple‐choice and open‐ended questions providing both quantitative and qualitative data (see Appendix C in the additional supporting information).
- Expert sampling of factors influencing a complex intervention for pre‐ and post‐operative voice therapy for BVFLs (White & Carding, 2020). A total of 10 purposively sampled expert voice therapists (mean of 22 years of practice, range = 7–38 years) were asked to describe factors influencing their current practice when working pre‐ and post‐operatively with patients with BVFLs and to outline views on optimum treatment pathways for this population. Interviews were recorded and transcribed verbatim. This data set comprised qualitative data, with rich insights into clinical practice and reasoning including information regarding the content, timing and intensity of the intervention delivered.
- PPI conversations. Nine conversations with five patients who had lived experience of BVFLs comprised the final source of evidence for this triangulation. Patient involvement activities were funded by a research design service PPI grant and included unstructured conversations, semi‐structured interviews and email correspondence. Two male and three female patients (aged 20–50 years) who had undergone phonosurgery were prompted to share their experience of surgery and voice therapy, and in semi‐structured interview specifically asked questions about the acceptability of voice therapy interventions and strategies to improve adherence and motivation. Involvement activities play a key role in shaping and influencing intervention development research (Tomlinson et al., 2019).

### TIDieR framework

Findings are presented according to the TIDieR framework (see Appendix C in the additional supporting information for full framework). Table 3 summarizes the convergence coding findings, showing areas of agreement (A), dissonance (D) and silence (S), together with the overall convergence assessment for each component. Further supplementary findings, including quotations to support each decision, are available in Appendix B in the additional supporting information.

- *TITLE*: Pre‐ and post‐operative voice therapy for BVFLs (PaPOV).
- *WHY*: Data sources supported the overall goal of a PaPOV: ‘To improve the voice and quality‐of‐life outcomes for individuals undergoing surgery for BVFLs.’

The intervention is built on four theoretical principles: wound healing; pre‐habilitation; motor learning principles; and behaviour change theory. The triangulation process found varying levels of agreement relating these theoretical principles to this specific population:

○ Wound healing versus mobilization. There is dissonance in the literature about periods of absolute voice rest to optimize wound healing following phonosurgery with a modal length of 7 days but a range from 2 to 21 days (White et al., 2021). This differed further with expert clinician and PPI interviews who generally advocated for 2–3 days absolute voice rest followed by early reintroduction of limited voicing. The benefits of early mobilization in relation to healing were recognized by the experts interviewed (White & Carding, 2020).
○ Pre‐habilitation. The importance of pre‐operative intervention was discussed in all sources of evidence. The published evidence suggests better outcomes for patients who receive pre‐operative intervention compared with those who receive post‐operative care only (Tang & Thibeault, 2017). Expert voice therapists were strongly in favour of identifying and managing factors contributing to the development of the lesion prior to surgery (White & Carding, 2020). PPI accounts corroborated the benefits of pre‐operative involvement, which helped manage personal and occupational expectations. A majority (65%) of clinician survey respondents indicated a preference for offering pre‐operative intervention and indicated that limited resources prevented this figure from being higher (White, 2022).
○ Motor learning principles. Voice therapy involves the acquisition of new vocal skills and habits, and the principles of motor learning and exercise physiology underpin the techniques used in the teaching and learning of coordinated physical movements such as voice production. Motor learning principles were explicitly addressed in the interview study, with participants linking dosage recommendations in home exercise practice to exercise physiology theory (White & Carding, 2020). Frequency, intensity and dosage information was extracted where available in the systematic review (White et al., 2021) but a lack of information in reporting practices meant that only two studies gave instructions on dosing of exercises with no accompanying rationale.
○ Behaviour change theory. Voice therapy is a behavioural intervention requiring patients to commit to behaviour change. Interview data and PPI discussions highlighted the value of providing education and advice in an individualized manner, which improved the patient's capabilities, opportunities, and motivation to change behaviours. The systematic review stated that addressing factors to improve adherence and compliance were critical components in any complex intervention but acknowledged that it was outside the scope of the review to explore these aspects.

*WHAT: MATERIALS*: Delivery of a complex voice therapy intervention for patients with BVFLs requires materials such as an intervention manual, access to electronic intervention content, peer support between clinicians delivering the intervention and support from intervention developers (Eldridge et al., 2016a; Hoffmann et al., 2014; Van Stan et al., 2019). A small number of the systematic review studies referred to an intervention manual, but this aspect was largely unexplored. PPI members further emphasized the need for a range of physical and electronic materials and having direct access to the speech and language therapy department for personal advice and reassurance. Expert voice therapists agreed that information and advice should be provided in both verbal and written formats.

- *WHAT: PROCEDURES*:

**TABLE 3:** Convergence coding and convergence agreement for components of a pre‐ and post‐operative voice therapy intervention (PaPOV) for benign vocal fold lesions (BVFLs)

			Sources of evidence				
TIDieR item	Brief description		SR	Survey	Interview	PPI	Convergence assessment yes/no^a^
**Title**	Pre‐ and post‐operative voice therapy for BVFLs		A	A	A	A	Y
**Why** – Theoretical principles and intervention goal	Wound healing and mobilization		D	S	A	A	N
	Pre‐habilitation literature		A	S	A	A	Y
	Exercise physiology theory		S	S	A	S	N
	Behaviour change theory		S	S	A	A	N
	Goal: ‘to improve voice and QOL outcomes for individuals undergoing surgery for BVFLs’		A	A	A	A	Y
**What** – Materials: provider	Use of an intervention manual		S	S	S	S	N
	Electronic access to intervention content		S	S	S	S	N
	Ongoing peer support between clinicians		S	S	S	S	N
	Support from intervention developers as required		S	S	S	S	N
**What** – Materials for participants/patients	Written information on the Intervention content for patients		S	S	A	A	N
	Clinician's contact details		A	S	A	A	Y
	Leaflets outlining education/information including voice care, voice production, BVFLs		A	S	A	A	Y
	Pre‐operative preparatory voice care advice sheets		S	A	A	A	Y
	Post‐operative voice use guide		S	S	A	A	N
	Written and/or video resources for exercises		S	S	A	A	N
	Personalized goal‐setting sheet		S	S	A	A	N
	Record sheet for home exercise practice		S	S	A	A	N
**What** – Procedures: assessment	Pre‐operative voice assessment procedures		A	A	A	A	Y
	Post‐operative voice assessment procedures		A	A	A	A	Y
**What** – Procedures: rehabilitation	Provide volitional ingredients	Information to enhance capabilities, opportunities and motivation to change behaviour	A	S	A	A	Y
	Provide vocal hygiene information	Actions/activities/substances: including level of voice use, hobbies, reflux	A	A	A	A	Y
		Diagnosis, anatomical and physiological changes related to BVFL	A	S	A	A	Y
		Treatment and prognosis	A	S	A	A	Y
	Practice sensory discrimination	Volume: develop skills to detect and monitor volume changes	S	S	A	S	N
		Quality of voice: develop skills to detect roughness breathiness and strain	S	S	A	A	N
		Vibrotactile sensation: develop skills to detect changes in vocal tract resonance	S	S	A	A	N
	Modify level of voice use	Instruction to adhere to absolute voice rest for *n* days	D	S	A	D	N
		Provide opportunities to practice confidential voicing	A	S	A	A	Y
		Resume relative voice rest within 1 week	D	S	A	A	N
	Provide opportunities to practice modified levels of muscle activation	Address compensatory muscle tension dysphonia pre‐operatively using direct therapy	A	A	A	S	Y
		Use direct techniques to address continued muscle tension dysphonia post‐operatively	A	A	A	A	Y
		Practice voicing without hard glottal attack pre‐operatively	A	A	A	S	Y
		Practice voicing without hard glottal attack post‐operatively	A	A	A	A	Y
		Practice projection techniques post‐operatively	A	S	S	A	N
		Practice pitch glides in the early post‐operative period	S	S	A	A	N
	Provide opportunities to practice voicing	Practice resonant voice exercise with forward placement	A	S	A	A	Y
	Provide semi‐occluded vocal tract exercises (SOVTE)	SOVTE using an external vehicle (tubing, straw, kazoo, flowball device)	S	S	D	D	N
		SOVTE using an anatomical structure (lips, tongue, voiced fricatives, nasal consonants)	A	S	A	A	Y
	Provide opportunities to practice breathing	Practice breathing techniques to improve the coordination of breath and voice	A	S	A	S	N
		Practice diaphragmatic breathing and breath control exercises	S	S	A	S	N
	Provide Amplification	Provide amplification to increase the voice signal volume in specific situations	S	S	S	S	N
	Apply pressure	Apply pressure consistent with a described proforma of manual therapy	S	S	A	S	N
	Provide opportunities to practice posture	Work on posture and alignment relevant to optimum positions for voicing	A	S	A	S	N
**Who** – Procedures: assessment	Patients undergoing phonosurgery, excluding malignant diagnoses, and vocal fold nodules		A	A	A	S	Y
	Qualified speech and language therapy (SLT)/SLP or equivalent with experience in voice disorders		A	A	A	S	Y
**How**	Motivational strategies including goal‐setting, problem solving, analogies, prompts cues		S	S	A	A	N
	Continuous assessment informs pace and direction of hierarchical task choice		A	A	A	S	Y
	Teaching the patient to monitor, analyse and alter their vocal production		A	S	A	A	Y
	Provide feedback	Continuous analysis of patient performance to inform feedback	S	S	A	S	N
	Use of multimodal feedback mechanisms to assist progress		S	S	A	S	N
	Use of augmented feedback tools, for example Laryngeal endoscopy		S	S	A	A	N
**Where**	At ENT SLT clinic setting		A	A	A	A	Y
	With practice at home and in functionally relevant situations, e.g., work, social settings for generalization		A	A	A	A	Y
**When**	Timing: benefits of pre‐operative intervention		A	A	A	A	Y
	Timing: benefits of post‐operative intervention		A	A	A	A	Y
	Dosing of exercises	Consistent and prescribed dose of exercises	D	D	D	D	N
		Individually tailored dose of exercises	S	S	A	A	N
		Frequent short episodes of home practice	S	S	A	S	N
	Number of sessions	Fixed number of pre‐ and post‐operative sessions	D	D	D	D	N
		Minimum standard with additional sessions as required	S	D	A	A	N

*Note*: ^a^Based on criteria explained in Table 2.

SR, systematic review; Survey, national survey of current practice; Interview, expert interview study; PPI, patient and public involvement activities; A, agreement; D, dissonance; S, silence; QOL, quality of life.

*Assessment procedures*: Findings across all data sources supported the use of multidimensional assessment tools both pre‐ and post‐operatively. Assessment should capture the impact of the voice disorder encompassing impairment, activity, participation and well‐being, along with exploration of predisposing, precipitating and perpetuating factors. A multidimensional assessment approach was used by 33/35 of the included papers in the systematic review, discussed by all interview participants and supported by PPI members who felt there was benefit in a range of perceptual, acoustic, endoscopic and patient‐reported assessment tools.

*Rehabilitation procedures* were divided into 11 components, with further subdivision of activities or ingredients. This was based broadly on terminology outlined in the RTSS for voice disorders (Van Stan et al., 2021).

The value of indirect therapy intervention was associated with high levels of agreement across all data sources. There was consensus for the inclusion of information, including education and advice on vocal activities, substances likely to cause inflammation, factors contributing to lesions development, anatomical and physiological changes, the treatment pathway and prognosis. This information was provided with the target of enhancing capabilities, opportunities and motivation to change behaviours.

Some direct therapy components achieved agreement in three sources of evidence and therefore met the stability of consensus criteria. These included providing opportunities to practice modified levels of muscle activation during phonation, using techniques to reduce compensatory muscle tension patterns and reduce hard glottal attack, and providing opportunities to practice voicing using resonant voice exercises with a focus on forward placement.

There was agreement that the PaPOV intervention should provide semi‐occluded vocal tract exercises (SOVTE) with an *anatomical vehicle* (e.g., lip or tongue trills, voiced fricatives, nasal consonants or semi‐occluded vowels). However, PPI discussions highlighted the potential for reduced compliance when *an external vehicle* such as tubing for water resistance therapy, a straw or kazoo was needed to undertake exercises. Experts disagreed amongst themselves on this issue and consequently this was regarded as an example of dissonance in the data sources.

The role of sensory discrimination in the PaPOV intervention had insufficient agreement to achieve consensus, but PPI and expert interview data was complimentary. Developing the skills to be able to detect changes in voice quality, volume and vocal tract resonance were seen as important precursors to being able to accurately perform and sustain a target voice. Providing opportunities to practise pitch glides, breathing techniques, manual therapy, postural modifications and projection as components of the intervention all had agreement in the rich expert interview data, but consensus was not achieved due to silence in other data sets.

- *WHO*: The PaPOV intervention is intended for those with benign lesions affecting the vibrating portion of the vocal folds. This excludes bilateral vocal fold nodules as surgery is rarely required in this population. There was agreement that this is a subgroup of patients with voice disorders, requiring specific considerations and that the intervention should be delivered by a qualified clinician with specific training in voice disorders. Expert voice therapists felt that these patients had a distinct treatment pathway with its own challenges, considerations and complexities. A large majority (33/35) of the studies in the systematic review reported exclusively on BVFL participants and excluded other pre‐malignant and malignant vocal fold lesions. This provided further supporting evidence for differing clinical pathways in benign and malignant lesion management. PPI members described elements of their voice therapy (e.g., voice rest and wound healing) which were specific to the BVFL patient population only.
- *HOW*: Mechanisms to deliver the intervention were variably discussed and included components such as the use of multimodal feedback techniques, motivational strategies and access to endoscopy for biofeedback. No evidence of dissonance was present in the data sources, but stability of consensus was poorly achieved due to silence in systematic review and survey data.
- *WHERE*: All data sources referred to delivery of face to face voice therapy sessions at the speech and language therapy (SLT) clinic with supplementary practice at home and in functional situations to enable carryover. There was no reference to ‘virtual’ or ‘online’ modes of delivery in any of the data sources.
- *WHEN*: The benefits of delivering voice therapy both before and after surgery were widely acknowledged and discussed in the ‘why’ section of results above.

The number of sessions in the PaPOV intervention did not achieve consensus. There was an overall trend in the survey data for patients to be offered two to four post‐operative sessions but pre‐operatively, there was widespread variation. One survey participant ‘strongly objected’ to a set number of sessions whereas another acknowledged a fixed pattern. Systematic review studies offered between one (Kaneko et al., 2017; Kiagiadaki et al., 2015) and 30 (Macedo et al., 2014) sessions (e.g., with dissonance in the data between those who offered a fixed number of sessions and those who tailored sessions according to individual need. PPI comments supported the need for flexibility in the number of sessions offered.

The dosing or frequency of exercises, both within sessions and in home practice, remains unclear and was seldom reported in the data sources. Where discussed, there was reference to exercise physiology and principles of motor learning, including repetition and frequency of practice (White & Carding, 2020).

### Summary of the results

In total, the intervention matrix detailed 61 possible components of the PaPOV intervention. Of these, 27 were categorized as having stability of consensus according to a priori criteria. A total of 34 failed to meet the criteria. This was more frequently due to silence (27) rather than dissonance (seven) in the data. Convergence coding and agreement summaries are presented in Table 3 with supporting quotes for the consensus assessment decision in the Appendix B in the additional supporting information.

## DISCUSSION

This study used a robust triangulation process from four data sources to inform the development of the PaPOV intervention for adults with BVFLs. By evidencing areas of agreement and stability of consensus across data sources, the validity of individual study findings has been enhanced. The included data sources approached the research question from different angles, giving the potential for a broader and more in‐depth consideration of the intervention.

The PaPOV intervention was described using all items from the TIDieR guidelines. Findings from the four data sources were used to populate a triangulation matrix to synthesize findings and assess convergence for each component of the intervention. Formal criteria from the TIDieR checklist (Hoffmann et al., 2014) and the Criteria for Reporting the Development and Evaluation of Complex Interventions (CReDECI) (Möhler et al., 2015) were followed during this intervention development work. All categories of intervention development described by the MRC framework for developing and evaluating complex interventions (Skivington et al., 2021), including theory, evidence and modelling, were included. Furthermore, the triangulation methodology undertaken in this study followed an established protocol to mitigate against potential methodological limitations (Booth et al., 2018; Farmer et al., 2006). Criteria for measuring the stability of convergence across data sources was set a priori improving rigour. The data sets triangulated in this study comprise mixed methods, but with significant volumes of qualitative data. Decisions regarding convergence coding are more nuanced. Regular discussion and full agreement within the study team limited the potential for bias. Furthermore, in keeping with qualitative methodology, coding was supported by direct quotes to illustrate and justify decision making.

### What information have we gained?

Based on this study we have a clearer idea of components which may form a best practice PaPOV intervention. Agreement and convergence in data sources helps to clarify the ‘overall goal’ of the intervention, which is to improve both the voice and quality of life of patients who are undergoing phonosurgery. Furthermore, there is consensus that patients undergoing phonosurgery should be offered intervention both before and after their surgery. Findings of this study emphasize the need for voice assessment to be multifactorial, that is, including both patient and clinician reported measures of voice and quality of life, and where possible including objective acoustic measures of voice.

Regarding the content of the intervention, agreement around the provision of indirect therapy (advice and education) is strong and has helped to identify keys bits of information, which are likely to be important for our patients. Specifically, pre‐operative voice therapy should include information about the diagnosis, and the impact of this on the patient's voice production and throat symptoms, their upcoming surgical procedure and the likely prognosis, including expected timescales for recovery. There is agreement that clinicians should provide information and develop shared goals regarding the patient's level of voice use, the impact of hobbies and the resumption of these post‐operatively, and the management of any reflux disease.

Consensus also surrounds the need to address compensatory muscle tension patterns, pre‐operatively using direct therapy, and where this fails to resolve spontaneously, this should be a continued area of focus following surgery. There is agreement that clinicians should work specifically with patients to teach patterns of phonation which minimize phonotrauma, such as focusing on reducing hard glottal attack, teaching resonant voice with forward placement and providing SOVTE. This information can provide the foundation for many components of best practice in pre‐ and post‐operative voice therapy.

### Where are the key uncertainties?

Convergence was not achieved in several components of the intervention and therefore some questions remain. The study exposed specific areas of the intervention which lacked agreement owing to silence or dissonance in the data sources included. One notable area of silence related to the provision of materials for providers of the intervention (TIDieR item: What – Materials). There was a failure (except for two) of the systematic review studies to include detail regarding intervention materials and this topic was outside the remit of the other sources of evidence. Although the included data sources contributed little to this aspect of the intervention, formal intervention development and reporting guidelines (Eldridge et al., 2016b; Hoffmann et al., 2014; Van Stan et al., 2019) support the inclusion of an intervention manual, electronic access to intervention content, ongoing peer support between clinicians and support from intervention developers as required. Where silence existed in the evidence from the systematic review, this was because of poor reporting practices in the included papers and a lack of detail on the materials provided, intervention components and detail on dosing of ingredients. This reinforces criticisms regarding the quality of intervention descriptions in behavioural interventions (Benninger, 2011; Hoffmann et al., 2013) and supports the call for improved descriptions of interventions to allow replication in both clinical and research settings, and enable robust evidence synthesis (Hoffmann et al., 2014). The nature and scope of the survey also led to frequent silences regarding specific intervention components (TIDieR item 4 What – Procedures). This may be explained by the use of broad questioning, eliciting only overarching information about the content of the intervention and/or the sampling strategy which included participants with limited experience of BVFL management. PPI members’ conversations gave important contributions to many, but not all items in the TIDieR framework. The interview data with expert voice therapists gave the most in‐depth interrogation into the components of the intervention. High levels of clinical expertise and in‐depth discussions give credibility to many components which showed agreement. However, in a number of cases, there was insufficient agreement in other sources to reach the stability of agreement as defined in Table 2.

Two key areas of dissonance related to wound healing and dosing of exercises. There is a considerable body of literature relating to the underlying theoretical principles of wound healing (Branski et al., 2006; Ishikawa & Thibeault, 2010; Kiagiadaki et al., 2015) and vocal fold scar (Allen, 2010; Friedrich et al., 2013) but varied recommendations in practice (White et al., 2021). Considerable work is required to identify how these principles should be directly applied to post‐operative exercise recommendations for patients with BVFLs (Verdolini Abbott et al., 2012; Whitling et al., 2018). Similarly, literature outlines theoretical principles of exercise dosing (Bergan, 2010) based on motor learning principles, but, again, it is unclear how this is related to the treatment of BVFLs. There is growing evidence for the positive effect of ‘boot camp’ style voice therapy (Patel et al., 2011) and massed practice (Bergan, 2010) but this has limited applicability in the immediate post‐operative period. Therefore, further exploration is required regarding how the principles of motor learning theory should influence a PaPOV intervention.

## IMPLEMENTATION AND RECOMMENDATIONS

This study has highlighted the need for further intervention development work and illuminated those areas which need continued scrutiny. This has been an important step in the process of developing a robust intervention and draws upon published guidance and recommendations. This research study should be viewed in the context of the MRC's intervention development guidance, with further research required to explore the PaPOV intervention, including the issue of tailoring and context which are essential in all complex interventions. Few intervention development studies are published, leading to uncertainty in the required steps from intervention development to intervention testing, recurrent pitfalls and research waste from developing interventions that never impact on healthcare (Booth et al., 2018; Hoddinott, 2015; Wight et al., 2016). An improved understanding of the detailed processes involved in the development of complex interventions through worked examples such as this, help to make these steps more transparent.

Currently, the PaPOV intervention is not suitable to be implemented into clinical practice. In its current form, it can be used to highlight components to consider when delivering pre‐ and post‐operative voice therapy. It can also be used as a methodological example for intervention development processes in other clinical specialisms. The protocol needs further refinement, prior to testing the feasibility of delivering the intervention. An international Delphi study is now underway to gain consensus on the outstanding questions related to the PaPOV intervention including. Once, finalized the protocol will be tested using a feasibility trial. This work is part of an ongoing NIHR‐funded Programme Grant.

## Supporting information

Supporting information

Supporting information

Supporting information

## Data Availability

The data that support the findings of this study are available from the corresponding author upon reasonable request.
