# Supplementary material for: Pre‐ and post‐operative voice therapy (PaPOV): Development of an intervention for patients with benign vocal fold lesions
Source: Int J Lang Commun Disord. 2022 Sep 1;58(1):94–110. doi: 10.1111/1460-6984.12771 (PMC10086784; doi:10.1111/1460-6984.12771)
Supplement: Supplementary file 1 — Supporting information [file JLCD-58-94-s001.docx]

**APPENDIX A:**

**The TIDieR (Template for Intervention Description and Replication) Checklist completed for the PaPOV intervention:**

| **TIDieR Item** | | **Pre and Post-operative voice therapy intervention (PaPOV) Brief description of components** | |
| --- | --- | --- | --- |
|  |  |  |  |
| **1)** | **BRIEF NAME:** Provide the name or a phrase that describes the intervention | Pre- and Post-operative voice therapy for benign vocal fold lesions | |
| **2)** | **Why**: Describe any rationale, theory, or goal of the elements essential to the intervention | Wound healing and mobilisation | |
|  |  | Prehabilitation literature | |
|  |  | Exercise Physiology Theory | |
|  |  | Behaviour Change Theory | |
|  |  | Goal – “to improve voice and QOL outcomes for individuals undergoing surgery for BVFLs”. | |
| **3)** | **What**: Materials: Describe any physical or informational materials used in the intervention, including those provided to participants or used in intervention delivery or in training of intervention providers. Provide information on where the materials can be accessed (e.g. online appendix, URL). | Use of an intervention manual | |
|  |  | Electronic access to intervention content | |
|  |  | Ongoing peer support between clinicians | |
|  |  | Support from intervention developers as required | |
|  |  | Written information on the Intervention content for patients | |
|  |  | Clinician’s contact details | |
|  |  | Leaflets outlining education/ Information including voice care, voice production, BVFLs | |
|  |  | Pre-operative preparatory voice care advice sheets | |
|  |  | Post-operative voice use guide | |
|  |  | Written and/or video resources for exercises | |
|  |  | Personalised goal setting sheet | |
|  |  | Record sheet for home exercise practice | |
| **4)** | **What:** Procedures: Describe each of the procedures, activities, and/or processes used in the intervention, including any enabling or support activities. | Pre-operative voice assessment procedures | |
|  |  | Post-operative voice assessment procedures | |
|  |  | Provide volitional ingredients | Information to enhance capabilities, opportunities and motivation to change behaviour |
|  |  | Provide Vocal hygiene information | Actions/Activities/Substances – including level of voice use, hobbies, reflux |
|  |  |  | Diagnosis, anatomical and physiological changes related to BVFL |
|  |  |  | Treatment and prognosis |
|  |  | Practice sensory discrimination | Volume – develop skills to detect and monitor volume changes |
|  |  |  | Quality of voice – develop skills to detect roughness breathiness and strain |
|  |  |  | Vibrotactile sensation – develop skills to detect changes in vocal tract resonance |
|  |  | Modify level of voice use | Instruction to adhere to absolute voice rest for *n* days |
|  |  |  | Provide opportunities to practice confidential voicing |
|  |  |  | Resume relative voice rest within 1 week |
|  |  | Provide opportunities to practice modified levels of muscle activation | Address compensatory MTD pre-operatively using direct therapy |
|  |  |  | Use direct techniques to address continued MTD post-operatively |
|  |  |  | Practice voicing without hard glottal attack pre-operatively |
|  |  |  | Practice voicing without hard glottal attack post-operatively |
|  |  |  | Practice projection techniques post-operatively |
|  |  |  | Practice pitch glides in the early post-operative period |
|  |  | Provide opportunities to practice voicing | Practice resonant voice exercise with forward placement |
|  |  | Provide semi-occluded vocal tract exercises (SOVTE) | SOVTE using an external vehicle (tubing, straw, kazoo, flowball device) |
|  |  |  | SOVTE using an anatomical structure (lips, tongue, voiced fricatives, nasal consonants) |
|  |  | Provide opportunities to practice breathing | Practice breathing techniques to improve the co-ordination of breath and voice |
|  |  |  | Practice diaphragmatic breathing and breath control exercises |
|  |  | Provide Amplification | Provide amplification to increase the voice signal volume in specific situations |
|  |  | Apply pressure | Apply pressure consistent with a described proforma of manual therapy |
|  |  | Provide opportunities to practice posture | Work on posture and alignment relevant to optimum positions for voicing |
| **5)** | **Who provided:**  For each category of intervention provider (e.g. psychologist, nursing assistant), describe their expertise, background and any specific training given | Patients undergoing phonosurgery, excluding malignant diagnoses, and vocal fold nodules | |
|  |  | Qualified SLT/SLP or equivalent with experience in voice disorders | |
| **6)** | **HOW:** Describe the modes of delivery (e.g. face-to-face or by some other mechanism, such as internet or telephone) of the intervention and whether it was provided individually or in a group. | Motivational strategies including goal setting, problem solving, analogies, prompts cues | |
|  |  | Continuous assessment informs pace and direction of hierarchical task choice | |
|  |  | Teaching the patient to monitor, analyse and alter their vocal production | |
|  |  | Provide Feedback | Continuous analysis of patient performance to inform feedback |
|  |  |  | Use of multimodal feedback mechanisms to assist progress |
|  |  |  | Use of augmented feedback tools eg. Laryngeal endoscopy |
| **7)** | **Where:** Describe the type(s) of location(s) where the intervention occurred, including any necessary infrastructure or relevant features | At ENT SLT Clinic setting | |
|  |  | With practice at home and in functionally relevant situations e.g. work, social settings for generalisation | |
| **8)** | **WHEN and HOW MUCH:** Describe the number of times the intervention was delivered and over what period of time including the number of sessions, their schedule, and their duration, intensity or dose | Timing – Benefits of pre-operative intervention | |
|  |  | Timing – Benefits of post-operative intervention | |
|  |  | Dosing of exercises | Consistent and prescribed dose of exercises |
|  |  |  | Individually tailored dose of exercises |
|  |  |  | Frequent short episodes of home practice |
|  |  | Number of sessions | Fixed number of pre and post-operative sessions |
|  |  |  | Minimum standard with additional sessions as required |
| **9)** | **TAILORING:** If the intervention was planned to be personalised, titrated or adapted, then describe what, why, when, and how. | Tailoring of;  -exercise dosing,  -level of complexity of tasks,  -number of sessions | |
| **10) ǂ** | **MODIFICATIONS:** If the intervention was modified during the course of the study, describe the changes (what, why, when, and how). | N/A | |
| **11)** | **HOW WELL:** Planned: If intervention adherence or fidelity was assessed, describe how and by whom, and if any strategies were used to maintain or improve fidelity, describe them. | N/A | |
| **12) ǂ** | **HOW WELL:** Actual. If intervention adherence or fidelity was assessed, describe the extent to which the intervention was delivered as planned | N/A | |

ǂ If completing the TIDieR checklist for a protocol, these items are not relevant to the protocol and cannot be described until the study is complete
