# Supplementary material for: Pre‐ and post‐operative voice therapy (PaPOV): Development of an intervention for patients with benign vocal fold lesions
Source: Int J Lang Commun Disord. 2022 Sep 1;58(1):94–110. doi: 10.1111/1460-6984.12771 (PMC10086784; doi:10.1111/1460-6984.12771)
Supplement: Supplementary file 2 — Supporting information [file JLCD-58-94-s003.docx]

**APPENDIX B: Triangulation Matrix**

**Triangulation Matrix, showing intervention components, sources of evidence, stability of consensus and examples/evidence to support decision making**

| **Intervention component**  **Brief description** | | **Sources of Evidence** | | | | **Convergence assessment Y/N*** | **Examples of agreement/dissonance from data sources**** |
| --- | --- | --- | --- | --- | --- | --- | --- |
|  |  | **SR** | **Survey** | **Interview** | **PPI** |  |  |
| Pre- and Post-operative voice therapy for benign vocal fold lesions | | A | A | A | A | **Y** | **SR:** Only 2/35 studies included a small proportion of patients with pre-malignant or early malignancies -clear understanding that the benign population have a different pathway of management. **Survey:** all were able to discuss management of pre and post operative management as a separate group. **Interview:** All felt that there were specific elements relating to wound healing, diagnosis, and timing and intensity of intervention that had different implications because of their pre/post-operative status . |
| Wound healing and mobilisation | | D | S | A | A | **N** | **SR:** Dissonance. Differences of opinion in voice rest practices. **Interview:** Discussed by all e.g. P08 "There is research that shows that stem cell recovery and healing occur because you're using it, you're moving it and you're exercising the muscle" P01 "It's that trade off isn't it between letting it heal sufficiently so you don't cause scarring by causing extra trauma, as opposed to not starting early enough" **PPI:** Helen: Inner conflict in patient's views about how much to talk - balancing rest v's pragmatics of life. Dan: "I was told to rest for 2 days but I didn't talk for 1 week" |
| Prehabilitation literature | | A | S | A | A | **Y** | **SR:** All included studies delivering Pre-op VT reported improved outcomes. Tang and Thibeault specifically show better outcomes in those we pre-op SLT. **Interview**: All felt pre-op intervention was essential. **PPI:** James: reported two experiences of surgery - one without and then one with pre-op SLT. Able to manage own expectations, able to have informed conversations with employers. |
| Exercise Physiology Theory | | S | S | A | S | **N** | **Interview**: P03- "Little and often is the key," " unless you practice them on a regular basis they will never become automatic… this is not going to generalise the behaviour" P10 - "a little and often mantra" is our main mantra" "No more than a couple of minutes at a time" "Up to 10 times a day depending on the client" "we are starting to learn... how muscle memory is built" P08 |
| Behaviour Change Theory | | S | S | A | A | **N** | **PPI**: Discussed how information and education with specific and individualised examples helped to improve their capability, Opportunities &Motivation to adhere and comply. **Interview**: Discussed motivation and adherence. |
| Goal – “to improve voice and QOL outcomes for individuals undergoing surgery for BVFLs”. | | A | A | A | A | **Y** | **PPI:** Dan highlighting the need for therapy to address both vocal function and related QOL measures. "I've been surprised and shocked if I'm honest at the variations in quality of treatment, advice and information. The whole experience has left me quite depressed at times and wondering if I'll ever be able to sing again with a joyful and carefree heart"  **SR:** Use of multidimensional outcome measures including acoustic, patient reported and perceptual |
| Use of an intervention manual | | S | S | S | S | **N** | **SR:** A few studies detailed the intervention manual of treatment, but this was inconsistent |
| Electronic access to intervention content | | S | S | S | S | **N** | No discussion about the clinician's requirement for access to intervention content. |
| Ongoing peer support between clinicians | | S | S | S | S | **N** | No discussion |
| Support from intervention developers | | S | S | S | S | **N** | No discussion |
| Written information on the Intervention content for patients | | S | S | A | A | **N** | **SR:** No mention of delivery vehicle, just "patients were given…" **Interview:** All participants discussed use of handouts. **PPI:** Helen - discusses handouts both in a positive and negative frame. Good when specific, relevant and explained. Bad when too many at once and unable to apply relevance. |
| Clinician’s contact details | | A | S | A | A | **Y** | **Interview:** P07 "They have all a post op sheet which has our departmental number on it. And they are encouraged…to ring us" **SR:** Zietels^1^ talks about managing patient anxiety and expectation and having regular contact with treating team. **PPI** "Knowing who and how to get in touch (with SLT) was really important" "Having a known SLT already before surgery meant I was less anxious as I could contact them if I had questions. |
| Leaflets outlining education/ Information including voice care, voice production, BVFLs | | A | S | A | A | **Y** | **PPI:** Kirsty "If they could have sent me any handouts or sheets, then I would have known what to do." **Interview:** P06 "they get a written sheet of dos and don'ts, of what voice rest really is" P07 "We give it face to face and on a handout, which also has online recommendations of where to go and look" "The patient will never remember what you’ve said so you will have to give them something in writing so that they can go away and read it” |
| Pre-operative preparatory voice care advice sheets | | S | A | A | A | **Y** | **Survey**: Across diagnoses 56-94% gave indirect therapy at least some of the time. **Interview**: "Clinicians unanimously agreed that the identification and management of factors which may have contributed to the development of the lesion was best addressed prior to surgery.” **PPI:** James “It was incredibly important to me to feel like I knew what I should be doing.” |
| Post-operative voice use guide | | S | S | A | A | **N** | **SR:** Resumption of voice use "under the supervision of SLT" NOT specified further. **Interview:** P02 "we do have a handout we give to patients on voice care post-surgery" p08 "We know from clinical discussions that a large proportion of patients do very well with direction. And that’s our opportunity to start that direction." **PPI**: Helen –“I had clear steps on what to do each day and a two week programme with gradual building up of vocal tasks” Caroline -“I want to know what I can do to look after my voice after my operation. Work are supportive but I need to know what I should and shouldn't be doing." |
| Written and/or video resources for exercises | | S | S | A | A | **N** | **Interview:** P05 "I ask them to record the exercises while we are doing them on their mobile, or we record it on a CD and we give it to them…And I do ask them to play the recording and sit down and ask them to do the exercises as if we are sitting together 1:1 at least once a day.” |
| Personalised goal setting sheet | | S | S | A | A | **N** | **Interview:** P08 "what are the baselines? where are we up to? What’s important? What’s the requirements of *your* voice?" |
| Record sheet for home exercise practice | | S | S | A | A | **N** | **Interview:** P03 "I'll give them a tick sheet that they can record how many times they do them" |
| Pre-operative voice assessment procedures | | A | A | A | A | **Y** | **SR:** Some variation in timings but all included multidimensional assessment of voice pre-operatively. **PPI:** Patients reviewed a range of outcomes and felt there was benefit in all. Liked the questionnaires, even though some were long as it reflected their experiences well. |
| Post-operative voice assessment procedures | | A | A | A | A | **Y** | As above |
| Provide volitional ingredients | Information to enhance capabilities, opportunities and motivation to change behaviour | A | S | A | A | **Y** | **Interview**: Coding identified 108 individual references to compliance making it the most referred to code. P09 "When they understand, they are more likely to do it". P03 "I find that with some post op patients they get a period of real enthusiasm and buoyancy and then sometimes that wanes" **PPI**: Discussion and consensus on the role of patient's ownership and strategies to maximise compliance, adherence, and motivation. Dan - "I wanted to hear that the thing could be removed with a simple operation and all would be back to normal, but that's no good if it's potentially not true" Kirsty- " I wanted to know how I can take care of my voice following surgery" Dan " I had good motivation because of my job but that may be harder for non-professional voice users". Opportunities - "I did find it a little difficult at times to carry out some of the exercises at home...often for the daft reason of embarrassment. When you've got a house full of people, it can feel a bit awkward to be making strange sounds in the kitchen or wherever" |
| Provide Vocal hygiene information | Actions/Activities/Substances – including level of voice use, hobbies, reflux | A | A | A | A | **Y** | Good consensus that this should be an important component of treatment and evidence that vocal hygiene is effective (as a collective) but individually there is limited evidence (or even contradictory evidence) for some aspects of what consistently is recommended. **SR:** 19/35 gave detail of the indirect content included. **Survey**: 64-91% of respondents ALWAYS gave indirect therapy to patients pre-operatively. Post-operatively 69-82% ALWAYS gave indirect therapy. **Interview:** All participants described in detail their rationale for including a mix of indirect and direct therapy. P06 "Whether it’s reflux, alcohol intake, smoking, you name it, he wants it sorted out in the run up to surgery" P03 "it may be giving them some general advice about that (reflux), about hydration, anything to get that vocal tract in the best possible health before they have their operation” |
|  | Diagnosis, anatomical and physiological changes related to BVFL | A | S | A | A | **Y** | **PPI:** Dan "The actual mechanics of voice use could have been better explained. I don't think many people have a clue about how the voice is actually produced and a good understanding of the larynx and how all the bits move and work would have helped me massively. It's the context for all the exercises, etc, so it seems logical to me that if you can picture what's going on down there, you'll understand better the importance and relevance of what you're trying to achieve" **Interview:** P05 "So understanding and education is very very important because it really makes a great difference in their compliance, and of their acceptance" |
|  | Treatment and prognosis | A | S | A | A | **Y** | **SR:** 29/35 discussed voice rest requirements and expectations of patient. **Interview:** Participants describe how surgical technique, had a significant impact on the intervention and projected outcomes for a patient. This could be used to manage expectations of a patient. |
| Practice sensory discrimination | Volume – develop skills to detect and monitor volume changes | S | S | A | S | **N** | **SR:** Only one study specifically referred to working on volume with patients **Interview:** P10 - A patient's vocal skill and awareness of their voice will influence the duration of VT. Discussed natural vocal skill, ability to self-monitor and correct. |
|  | Quality of voice – develop skills to detect roughness breathiness and strain | S | S | A | A | **N** | **Interview:** P10 - A patient's vocal skill and awareness of their voice will influence the duration of VT. Discussed natural vocal skill, ability to self-monitor and correct. |
|  | Vibrotactile sensation – develop skills to detect changes in vocal tract resonance | S | S | A | A | **N** | **Interview**: P09 "And once they’ve felt that it’s easy again, normally they revert back to what they comfortably did" P10“Most of them need to learn to play it again” P05 "I ask a lot of questions along the therapy… How do you like this? How does it feel? Do you like this? Does it feel alien? DO you feel fine? Does it feel ok?" **PPI:** Helen - Critical to understand what I was aiming for with the exercises and to have a feeling or sound that I could latch onto, so I knew I was doing it right. |
| Modify level of voice use | Instruction to adhere to absolute voice rest for *n* days | D | S | A | D | **N** | **SR:** Wound healing theory supports 48 hours voice rest in line with re-epithelialisation. Minor differences favour shorter voice rest periods. Adherence literature comparing absolute and relative voice rest shows absolute voice rest (AVR) is rarely achieved, but participants on AVR vocalise less than those on relative voice rest. **Interview:** P10 "So they have 2 days total voice rest and then we advise them that as the voice comes back just use confidential voice and to use that for the next few days, gradually building up the amount of voice that they are using but no more than a few minutes at a time, no long conversations" **PPI:** Helen: Inner conflict in patient's views about how much to talk - balancing rest v's pragmatics of life. Dan: "I was told to rest for 2 days but I didn't talk for 1 week" |
|  | Provide opportunities to practice confidential voicing | A | S | A | A | **Y** | **SR:** Some evidence for use of confidential voice. Gradual reintroduction of vocal activities is discussed. **Interview:** P05 “2 weeks gentle voice use, still no phone calls, no talking above background noise, no going back to work if their work is vocally demanding, and it’s 10-15 minutes bursts of gentle voice in a quiet environment maximum of two hours a day and then they build up gradually" |
|  | Resume relative voice rest within 1 week | D | S | A | A | **N** | **SR: T**he average period of voice rest was 7 days, but some centres required 14 days absolute voice rest. **Interview/PPI:** see previous two rows re resumption of vocal activities |
| Provide opportunities to practice modified levels of muscle activation | Address compensatory MTD pre-operatively using direct therapy | A | A | A | S | **Y** | **Survey**: "We want to ensure compensatory behaviour is minimised so (it) doesn't carry over post-op" “(Direct therapy) mostly focuses on laryngeal deconstriction of compensatory muscle tension". >50% for all diagnoses would carry out pre-operative direct therapy. **Interview:** P05 "If any compensatory muscle tension is identified, during the voice assessment, then that is my main aim. Let’s undo the compensation first." P07 "Whether it was primary or secondary (MTD) who knows but you still need to address it because it’s there then in the laryngeal gesture” |
|  | Use direct techniques to address continued MTD post-operatively | A | A | A | A | **Y** | **SR:** Ten studies detailed the direct therapy techniques that they would carry out post operatively. **Survey:** >50% for all diagnoses would carry out direct therapy post-operatively. **Interview**: P04 "Really its then more using the voice, placing the speaking voice, easy onset, actually things targeting vocal tract tension. Err (pause) Tongue root tension, jaw alignment, all the basics again" |
|  | Practice voicing without hard glottal attack pre-operatively | A | A | A | S | **Y** | **SR**: 6 studies mentioned reducing vocal abuse. **Interview:** P10 "If they’ve got a lot of hard attack we might see if we can eliminate some of that first through soft onsets" **Survey:** >50% for all diagnoses would carry out direct therapy “(Therapy involves) minimising aggressive vocal habits that may have precipitated / perpetuated problem” |
|  | Practice voicing without hard glottal attack post-operatively | A | A | A | A | **Y** | **Interview:** P02 "I think so much tends to be hard glottal attack and voice abuse" **SR**: Six studies mentioned reducing vocal abuse. **Survey**: >50% for all diagnoses would carry out direct therapy post operatively. “Patients need to be able to readjust the muscular laryngeal behaviour with their optimised physiology” |
|  | Practice projection techniques post-operatively | A | S | S | A | **N** | **SR:** Four studies included functional tasks or ‘carry over exercises’ e.g. practice in varied environments in the post-operative description of voice therapy. **Interview:** No examples of discussion around safe shouting/projection **PPI:** Helen felt pressure afterwards and anxiety about doing too much too soon for fear of ruining her new voice. She found social situations stressful and felt this triggered bad/negative behaviours. |
|  | Practice pitch glides in the early post-operative period | S | S | A | A | **N** | **Interview:** P06 strongly advocates early glides. P04 "Then (day 4 onwards) we start to gradually extend the pitch range, but we’d practice that with them. So, you know, we’d say, “if you feel that there’s a bit more flexibility, you go there”. "If the patient was having some difficulty accessing the high range then I might well do some pitch glides, or sirens” P05 "So we will go again into the semi-occluded vocal tract exercises with the lip trills and the tongue rolls and we will glide from there and glide within a chest voice" P08 "I would use very gentle but very limited siren, so they are doing tilts and starting to get an idea of stretch and relax which is really important afterwards if you are going to stretch and relax scar tissue to create as much flexibility" |
| Provide opportunities to practice voicing | Practice resonant voice exercise with forward placement | A | S | A | A | **Y** | **SR:**4 studies specifically mentioned resonant voice therapy as an ingredient of direct therapy. **Interview**: P10 "I often get them doing humming, and I’ll work through flexibility trying to shift pitch and volume, just seeing how their voice responds." P03 "In that first session I’d be talking about ways of gradually coming back to it and ways of approaching it really with humming… just very gently" P05 “try humming with that and gliding with that, then the humming into two-word phrases and then we extend that from there." P04 "We quickly then move on to other type of voice work...resonant voice type techniques, humming if they need to." |
| Provide semi-occluded vocal tract exercises (SOVTE) | SOVTE using an external vehicle (tubing, straw, kazoo, flowball device) | S | S | D | D | **N** | **SR:** Only one study refers to tube phonation. **Interview:** P05 “I would rather them (be) doing something that doesn’t involve them sitting holding something with equipment because that will limit when they can do it” "Well lax Vox is quite tricky. Because if the patient is not doing it right then you are establishing an awful lot of tongue base tension. **PPI:** Dan "It was also tricky to carry out the 'tube and water' exercise for me at school and prior to gigs. I'd sometimes forget the tube or not have a glass of water handy…and it's also rather embarrassing explaining to people what's going on.” |
|  | SOVTE using an anatomical structure (lips, tongue, voiced fricatives, nasal consonants) | A | S | A | A | **Y** | **PPI:** Dan “I read up myself about the mechanics of the exercise and ended up substituting in lip trills as a way of creating pressure above and below the vocal cords. That seemed to work better for me." **SR:** Studies refer to exercise names e.g. vocal function and resonant voice without referring to SOVT exercises. **Interview:** Multiple references to anatomical SOVT exercises e.g. lip trills, accent method. P05 "I would usually use one of the semi-occluded vocal tract techniques. Whichever one of them would work with the patient, but these are usually my ‘go-to’ solution to re-establish the mucosal wave" “They can trill their tongue and they can trill their lips while they are washing the dishes and making their bed, so I would prefer giving them something that they can do on the go.” |
| Provide opportunities to practice breathing | Practice breathing techniques to improve the co-ordination of breath and voice | A | S | A | S | **N** | **SR:** 4 studies referred to breath support exercises, 1 flow phonation which draws on principles of co-ordinating breath and voice. **Interview:** P03 "I’d always be looking at breathing patterns at respiratory patterns, so I’d be drawing on accent method”. "I try very much in patients to under pin that open posture that gentle adduction with support from below." P05 "is it lack breath support, which almost all of them have" P08 "how they breathe and when they breathe. I’d work at that level first. |
|  | Practice diaphragmatic breathing and breath control exercises | S | S | A | S | **N** | **Interview**: P03 "I’d always be looking at breathing patterns at respiratory patterns” P08 “So it depends where they are with their co-ordination, how far back you need to go back through respiration and using it and body awareness" |
| Provide Amplification | Provide amplification to increase the voice signal volume in specific situations | S | S | S | S | **N** | Although discussed in the broader voice therapy literature, no discussion relating to amplification devices were evident in these data sources |
| Apply pressure | Apply pressure consistent with a described proforma of manual therapy | S | S | S | S | **N** | Although discussed in the broader voice therapy literature, no discussion relating to laryngeal manual therapy, manipulation, massage, or other similar therapy approaches were discussed in these data sources. |
| Provide opportunities to practice posture | Work on posture and alignment relevant to optimum positions for voicing | A | S | A | S | **N** | **SR:** 4 studies referenced the inclusion of therapy targeting posture or extrinsic laryngeal tension. **Interview:** P03 "I'd be having a look at posture, we’re trying to get the voice out now, so I’d be looking at the person as a whole and the pattern that they are showing me." |
| Patients undergoing phonosurgery, excluding malignant diagnoses, and vocal fold nodules | | A | A | A | S | **Y** | **Survey:** Different patterns of intervention for nodules v’s all other lesion types consistently reported **SR: :** Only 2/35 studies included a small proportion of patients with pre-malignant or early malignancies. Benign population have a different pathway of management and surgical principles |
| Qualified SLT/SLP or equivalent with experience in voice disorders | | A | A | A | S | **Y** | **Survey:** Sample was all SLTs, those with inexperience acknowledged difficulties of managing complex, less common lesions. **SR:** Where included, therapy was delivered by SLT/SLP **Interview:** Recruitment strategy focused on expert SLTs as they were deemed to be best placed to contribute |
| Motivational strategies including goal setting, problem solving, analogies, prompts cues | | S | S | A | A | **N** | **PPI**: Discussion and consensus on the role of patient's ownership and strategies to maximise compliance, adherence and motivation. **Interview:** Coding identified 108 individual references to compliance making it the most referred to code. Compliance may be high in this group as they see it as a short term investment but frequent references to problem solving, setting individualised goals. |
| Continuous assessment informs pace and direction of hierarchical task choice | | A | A | A | S | **Y** | **Interview:** A key patient factor was the vocal ability of the patient. This influenced the type of exercises prescribed by the clinician and the speed of progression through a hierarchy of exercises. Additional demands on professional voice users increased the pressure to rehabilitate quickly but ultimately this was driven by continuous assessment **SR:** Several studies referred to individualised programmes depending on success of achieving a target. |
| Teaching the patient to monitor, analyse and alter their vocal production | | A | S | A | A | **Y** | **PPI:** James - Some of the exercises were the same as 2014, but I felt that I was given a much better understanding of what was going on. Caroline -felt it was critical to understand what she was aiming for with the exercises and to have a feeling or sound that she could latch onto to show her that she was doing it right. Debs- was confident in what to do because she practiced these lots in the session with the therapist. |
| Provide Feedback | Continuous analysis of patient performance to inform feedback | S | S | A | S | **N** | **Interview:** P08 "What can they actually produce? Is it only producible with lip trill or is it only producible with bubble blowing for example or a resistant sound? ...Try and use some of those exercises. If they are struggling with those then I would go backwards to accent method and tongue base relaxation and then forward back into the vocal fold amplitude exercises" "A very experienced therapist is going to see that they are not achieving it or HEAR that they are not achieving it, or we might have the opportunity to do the therapy under a view” (endoscopy). |
|  | Use of multimodal feedback mechanisms to assist progress | S | S | A | S | **N** | **Interview** P05 "I really focus on patients feeling what they are doing rather than listening and achieving the ‘optimum voice’ because it doesn’t work that way and sometimes, I actually get them close their eyes and ignore that I’m there and try and feel and depend a lot on their tactile feedback." |
|  | Use of augmented feedback tools e.g. Laryngeal endoscopy | S | S | A | A | **N** | **Interview**: P05 "Pre-operatively (endoscopy) would be FABULOUS because there and then you would identify the laryngeal gesture that the patient is doing trying to compensate" "we actually also play that recording to the patient, not just so that they can see the pathology and understand that but so we can highlight what they are doing, trying to compensate” **PPI:** James "Being able to have the scope to see my vocal cords has also been a great advantage. Some of the exercises were the same but I felt that I was given a much better understanding of what was going on" Dan "Seeing the images of my throat on the screen.... it was a revelation. For someone like me who has a strong imagination, it was great just to be able to see what you're dealing with." |
| At ENT SLT Clinic setting | | A | A | A | A | **Y** | Data sources related to pre-COVID intervention. All Voice therapy was delivery in clinic settings |
| With practice at home and in functionally relevant situations e.g. work, social settings for generalisation | | A | A | A | A | **Y** | **SR:** 4 studies specifically referred to the inclusion of carry over activities. **Interview:** References to homework tasks, importance of using generalisable tasks, impacts of voice improvement on functional activities. **PPI:** Helen Initially found it had little relevance to her day to day life – e.g. how was blowing through a straw to help talking in the pub. Later intervention Helen felt that there was a more practical application of the exercises. Caroline- found social situations stressful and felt this triggered bad/negative behaviours. |
| Timing - Pre-operative intervention | | A | A | A | A | **Y** | Consensus in the potential benefits of delivering pre-operative intervention to patients across all key sources of evidence |
| Timing - Post-operative intervention | | A | A | A | A | **Y** | Consensus in the potential benefits of delivering post-operative intervention to patients across all key sources of evidence |
| Dosing of exercises | Consistent and prescribed dose of exercises | D | D | D | D | **N** | Wide differences on dosing recommendations reported across data sources. **SR**: Limited mention of dosing but individualised programmes referred to. **Interview:** Mix of prescriptive and individualised comments. P07 , “Generally, a little and often mantra is our main mantra.” P08 "I tell them how many times a day, how I want it spaced out. I’m very very control freak prescriptive I think (laughing). I think the clearer the instructions that you give to somebody the higher the chance that they will be able to follow them" |
|  | Individually tailored dose of exercises | S | S | A | A | **N** | **Interview:** We will encourage little and often in this department. Do we define what little is? No more than a couple of minutes at a time. Do we describe what ‘often’ is? Up to 10 times a day depending on the client so that’s probably as prescriptive as we get" **PPI:** Dan "I did a lot of my voice exercises in the car or while walking the dog which probably aren't ideal times but it worked for me" |
|  | Frequent short episodes of home practice | S | S | A | S | **N** | **Interview:** P08 "And then I would say, you need an element of repetition for your body and mind to learn the movements so little and often" |
| Number of sessions | Fixed number of pre and post-operative sessions | D | D | D | D | **N** | **Survey:** 60-80% would undertake 2-4 post operative sessions. "Very much depends on client's level of satisfaction with their voice and motivation" "I would strongly object to a set average number of sessions or length of treatment per condition as patients need different input and respond/recovery differently.” "Work on a 4 session model of delivery. **PPI** Dan "I was however, a bit disappointed that the NHS therapy seemed to have a limit to the number of sessions... understandable what with the pressures on the NHS." |
|  | Minimum standard with additional sessions as required | S | D | A | A | **N** | **Interview:** "Successful completion of voice therapy relates to an individual achieving a set of personalized functional goals rather than restoration of normal voice quality". Patients presented with individual requirements, needs and skills and these factors would inherently influence the intervention patients received pre and post-operatively. "Very much depends on clients level of satisfaction with their voice and motivation" "Entirely depends on the rest of the physiology" "Longer if psychological components influencing behaviours or environmental/ occupational challenges that restrict change in vocal habits / ability to apply recommendations" **PPI**: "I was however, a bit disappointed that the NHS therapy seemed to have a limit to the number of sessions… understandable what with the pressures on the NHS." Helen - felt that there should be a minimum offered with additional options for professional voice users, as required. |

SR = Systematic Review, Survey = National survey of current practice, Interview = Expert Interview study, PPI= Patient and Public Involvement activities

A = Agreement, D = Dissonance, S = Silence

* Based on criteria explained in Table 2.

**Further examples available on request by e-mailing corresponding author

1. Zeitels SM. The Art and Craft of Phonomicrosurgery in Grammy Award-Winning Elite Performers. *The Annals of otology, rhinology, and laryngology*. 2019;128(3_suppl):7S. doi:10.1177/0003489418810697
