# Supplementary material for: Pre‐ and post‐operative voice therapy (PaPOV): Development of an intervention for patients with benign vocal fold lesions
Source: Int J Lang Commun Disord. 2022 Sep 1;58(1):94–110. doi: 10.1111/1460-6984.12771 (PMC10086784; doi:10.1111/1460-6984.12771)
Supplement: Supplementary file 3 — Supporting information [file JLCD-58-94-s002.docx]

**APPENDIX C:**

**Further information on data sources**

**National Survey of Current Practice Questions**

1. **How often do you see the following groups of patients for voice assessment or therapy before phonosurgery? (This could include a Joint Voice Clinic or SLT setting).**

Comments:

|  | Always | Usually | Sometimes | Rarely | Never |
| --- | --- | --- | --- | --- | --- |
| Vocal fold nodules |  |  |  |  |  |
| Vocal fold polyp |  |  |  |  |  |
| Vocal fold cyst |  |  |  |  |  |
| Sulcus/mucosal bridge |  |  |  |  |  |
| Other benign vocal fold lesion (e.g. papilloma, granuloma, Reinke’s oedema) |  |  |  |  |  |

1. **When you see a patient PRE-OPERATIVELY, how often do you undertake INDIRECT therapy? (e.g. advice, information, education, strategies)**

|  | Always | Usually | Sometimes | Rarely | Never |
| --- | --- | --- | --- | --- | --- |
| Vocal fold nodules |  |  |  |  |  |
| Vocal fold polyp |  |  |  |  |  |
| Vocal fold cyst |  |  |  |  |  |
| Sulcus/mucosal bridge |  |  |  |  |  |
| Other benign vocal fold lesion (e.g. papilloma, granuloma, Reinke’s oedema) |  |  |  |  |  |

Comments:

1. **When you see a patient PRE-OPERATIVELY, how often do you undertake DIRECT therapy? (e.g. Therapy exercises and techniques)**

|  | Always | Usually | Sometimes | Rarely | Never |
| --- | --- | --- | --- | --- | --- |
| Vocal fold nodules |  |  |  |  |  |
| Vocal fold polyp |  |  |  |  |  |
| Vocal fold cyst |  |  |  |  |  |
| Sulcus/mucosal bridge |  |  |  |  |  |
| Other benign vocal fold lesion (e.g. papilloma, granuloma, Reinke’s oedema) |  |  |  |  |  |

Comments:

1. **For the following groups of patients, how many PRE-OPERATIVE sessions would you offer on average?**

|  | 0-1 | 2-4 | 5-8 | >8 |
| --- | --- | --- | --- | --- |
| Vocal fold nodules |  |  |  |  |
| Vocal fold polyp |  |  |  |  |
| Vocal fold cyst |  |  |  |  |
| Sulcus/mucosal bridge |  |  |  |  |
| Other benign vocal fold lesion (e.g. papilloma, granuloma, Reinke’s oedema) |  |  |  |  |

Comments:

1. **How often do you see a patient on the day of surgery?**

Comments:

|  | Always | Usually | Sometimes | Rarely | Never |
| --- | --- | --- | --- | --- | --- |
| Vocal fold nodules |  |  |  |  |  |
| Vocal fold polyp |  |  |  |  |  |
| Vocal fold cyst |  |  |  |  |  |
| Sulcus/mucosal bridge |  |  |  |  |  |
| Other benign vocal fold lesion (e.g. papilloma, granuloma, Reinke’s oedema) |  |  |  |  |  |

1. **How often do you see the following groups of patients for voice assessment or therapy POST-OPERATIVELY?**

|  | Always | Usually | Sometimes | Rarely | Never |
| --- | --- | --- | --- | --- | --- |
| Vocal fold nodules |  |  |  |  |  |
| Vocal fold polyp |  |  |  |  |  |
| Vocal fold cyst |  |  |  |  |  |
| Sulcus/mucosal bridge |  |  |  |  |  |
| Other benign vocal fold lesion (e.g. papilloma, granuloma, Reinke’s oedema) |  |  |  |  |  |

Comments:

1. **At what point do patients have their FIRST post-operative voice therapy appointment with a Speech and Language Therapist?**

Comments:

|  | Always | Usually | Sometimes | Rarely | Never |
| --- | --- | --- | --- | --- | --- |
| Vocal fold nodules |  |  |  |  |  |
| Vocal fold polyp |  |  |  |  |  |
| Vocal fold cyst |  |  |  |  |  |
| Sulcus/mucosal bridge |  |  |  |  |  |
| Other benign vocal fold lesion (e.g. papilloma, granuloma, Reinke’s oedema) |  |  |  |  |  |

1. **When you see a patient POST-OPERATIVELY, how often do you undertake INDIRECT therapy? (e.g. advice, education, information, strategies)**

Comments:

|  | Always | Usually | Sometimes | Rarely | Never |
| --- | --- | --- | --- | --- | --- |
| Vocal fold nodules |  |  |  |  |  |
| Vocal fold polyp |  |  |  |  |  |
| Vocal fold cyst |  |  |  |  |  |
| Sulcus/mucosal bridge |  |  |  |  |  |
| Other benign vocal fold lesion (e.g. papilloma, granuloma, Reinke’s oedema) |  |  |  |  |  |

1. **When you see a patient POST-OPERATIVELY, how often do you undertake DIRECT therapy? (e.g. therapy exercises and techniques)**

|  | Always | Usually | Sometimes | Rarely | Never |
| --- | --- | --- | --- | --- | --- |
| Vocal fold nodules |  |  |  |  |  |
| Vocal fold polyp |  |  |  |  |  |
| Vocal fold cyst |  |  |  |  |  |
| Sulcus/mucosal bridge |  |  |  |  |  |
| Other benign vocal fold lesion (e.g. papilloma, granuloma, Reinke’s oedema) |  |  |  |  |  |

Comments:

1. **For the following groups of patients, how many POST-OPERATIVE sessions would you give on average?**

Comments:

|  | 0-1 | 2-4 | 5-8 | >8 |
| --- | --- | --- | --- | --- |
| Vocal fold nodules |  |  |  |  |
| Vocal fold polyp |  |  |  |  |
| Vocal fold cyst |  |  |  |  |
| Sulcus/mucosal bridge |  |  |  |  |
| Other benign vocal fold lesion (e.g. papilloma, granuloma, Reinke’s oedema) |  |  |  |  |
